# Effective Binding of a Phosphatidylserine-Targeting Antibody to Ebola Virus Infected Cells and Purified Virions

**DOI:** 10.1155/2015/347903

**Published:** 2015-03-01

**Authors:** S. D. Dowall, V. A. Graham, K. Corbin-Lickfett, C. Empig, K. Schlunegger, C. B. Bruce, L. Easterbrook, R. Hewson

**Affiliations:** ^1^Public Health England, Porton Down, Wiltshire, Salisbury SP4 0JG, UK; ^2^Peregrine Pharmaceuticals, Inc., Tustin, CA 92780, USA

## Abstract

Ebola virus is responsible for causing severe hemorrhagic fevers, with case fatality rates of up to 90%. Currently, no antiviral or vaccine is licensed against Ebola virus. A phosphatidylserine-targeting antibody (PGN401, bavituximab) has previously been shown to have broad-spectrum antiviral activity. Here, we demonstrate that PGN401 specifically binds to Ebola virus and recognizes infected cells. Our study provides the first evidence of phosphatidylserine-targeting antibody reactivity against Ebola virus.

## 1. Introduction

The family Filoviridae includes two genera,* Ebolavirus *and* Marburgvirus.* The genus* Ebolavirus* includes five species (*Bundibugyo ebolavirus*,* Reston ebolavirus*,* Sudan ebolavirus*,* Taï Forest ebolavirus*, and* Zaire ebolavirus*).* Ebolavirus* strain Ebola (EBOV) is the only member of the* Zaire *species of* Ebolavirus* [1, 2]. EBOV first came to medical attention in 1976 with a disease outbreak in Zaire (now Democratic Republic of Congo (DRC)) [3]. Sporadic outbreaks of Ebola virus disease have occurred naturally since then, sometimes characterised by large epidemics, for example, in the town of Kikwit, DRC, in 1995 (315 cases and 244 deaths) [4]. Since 2001 epidemics have been occurring with increasing frequency which may be related to the increasing encroachment of human beings on tropical rain forests and once-isolated rural villages [5].

Human EBOV infection results in high lethality. Indeed, case-fatality rates of the African EBOV are as high as 90%, with no prophylaxis or treatment available. Consequently the virus is classified as a Risk Group 4 agent, mandating the use of high containment laboratory infrastructure for work with infectious materials. Further classification as a Category A Priority Pathogen by the US NIH/NIAID reflects concern of its potential use as a bioweapon [6]. New therapeutic strategies against EBOV infection are urgently required. Currently these range from antisense technology (chemically modified antisense oligonucleotides that interfere with the translation of viral mRNA) [7, 8] to therapeutic antibodies against specific EBOV proteins [9–11]. While these therapies rely on viral specific interactions, an alternative host-targeted antibody therapy enabling a broader viral specificity has recently gained favour. Bavituximab (PGN401) is a monoclonal human-mouse chimeric antibody. The Fv region was obtained from the mouse IgG3 monoclonal antibody 3G4 specific towards phosphatidylserine (PS) [12] which was subsequently joined to human IgG1*κ* constant regions [13]. In healthy cells, PS resides predominantly in the inner leaflet of the plasma membrane, where it is inaccessible to circulating antibodies, but translocates to the outer leaflet and externalizes upon cell injury or death [14]. Surface exposure of PS is then accompanied by cell death through apoptosis [15], mediated in part through recognition by T cell immunoglobulin mucin proteins [16]. PS exposure is now accepted as a ubiquitous phenomenon of apoptosis that is independent of cell type and the cell death-inducing trigger [17]. PGN401 was primarily used in mouse models of cancer, which have tumor vasculature with PS expression on endothelial cells [18]. It appears that 3G4 does not bind PS directly but through complexes of the PS-binding plasma protein *β*2-glycoprotein 1 (*β*2GP1) [19]. The antibody binds to PS-expressing membranes by crosslinking two molecules of *β*2GP1 bound to PS on the membrane [19]. PGN401 has successfully completed several clinical trials, including Phase I in patients with advanced solid tumors [20] and Phase II in patients with advanced breast cancer and non-small cell lung cancer (NSCLC) [13]. It is now entering Phase III trials for NSCLC [21]. Therefore, PGN401 is known to be safe in human studies and the pharmacokinetics of the antibody has been studied.

Surface exposure of PS antigen is also a consequence of viral infection through virus-induced apoptosis events. These also result in a loss of lipid asymmetry due to the translocation of PS from the inner to the outer layer of the infected cells' plasma membrane [22]. Antibodies binding the exposed PS appear to limit viral infection by initiating the removal of enveloped viruses from the bloodstream through the induction of antibody-dependent cellular cytotoxicity (ADCC) which ultimately eliminates virus infected cells [22, 23]. Whilst PS relocation is not the final step in apoptosis, cells expressing it are still likely to be actively producing virus so opsonizing them for ADCC may limit or slow the progression of infection. Major advantages of PGN401 over other antibody treatments against EBOV include specificity for infected cells and independence of virus escape mutations [24]. Antibodies are also attractive anti-infective therapeutics due to their exquisite specificity and their ability to recruit additional immune system components such as complement and natural killer cells, facilitating pathogen inactivation and removal [22]. Most of the antiviral work with PGN401 has been undertaken with hepatitis C virus [25] and has extended to clinical trials [26, 27]. In 2008, Soares et al. reported the efficacy of PGN401 in guinea pigs infected with Pichindé virus [23], a model that closely resembles Lassa fever in humans [28]. Therefore, it was hypothesized that PGN401 may also have therapeutic potential for other hemorrhagic fever viruses. In this report, we evaluate the* in vitro* efficacy of PGN401 to bind to EBOV virions and EBOV-infected cells.

## 2. Methods

### 2.1. Virus

EBOV isolate ME718 was used in this work. This was originally isolated during an outbreak in October 1976 [3] in Yambuku, Mongala Province, in what is currently the northern Democratic Republic of the Congo, and simultaneously reported in three publications [29–31]. Virus stocks used for this work were grown in VeroE6 cells (European Collection of Cell Cultures, UK) cultured in Leibovitz's L15 (L15) media containing 5% fetal calf serum (FCS), and aliquots were stored at −80°C. Virus titres were determined by 100-fold dilution with L15 media without any FCS added. 100 *μ*L of each dilution was overlaid onto semiconfluent cell monolayers in four replicate 12.5 cm^2^ tissue culture flasks and left to adsorb for 1 hour. A volume of 5 mL media was then added and cells were incubated at 37°C for 6-7 days. Cytopathic effects were observed using microscopy, and the results from each dilution were used to calculate 50% tissue culture infective dose (TCID_50_) using the Reed-Muench method [32].

### 2.2. Flow Cytometry Assay

VeroE6 cells in 12.5 cm^2^ tissue culture flasks were infected with EBOV at a multiplicity of infection (MOI) of approximately 0.5. After five days of infection, the media were removed and the cell monolayer washed with phosphate buffered saline (PBS). For staining with PGN401, the antibody was used at a concentration of 1 *μ*g/mL in flow cytometry buffer consisting of 10 mM HEPES pH 7.4 (Sigma, UK), 140 mM NaCl (Sigma, UK), and 2.5 mM CaCl_2_ (Sigma, UK) with 50% FCS (Invitrogen, UK). To each flask, 1 mL antibody suspension was added and left on ice for 30 minutes. Unbound antibody was removed by washing with PBS, and cells detached using TrypLE Express solution (Invitrogen, UK) with incubation at 37°C to aid enzymatic activity. Once detached, the cell suspension was transferred to cell culture tubes used for flow cytometry staining. Cells were washed twice with flow cytometry buffer by addition of 2 mL buffer and centrifugation at 400 ×g for 5 minutes. Anti-EBOV antibody (clone FE25, Lifespan Biosciences, USA) was diluted to 100 *μ*g/mL in flow cytometry buffer containing 50% FCS, and 100 *μ*L was added to each cell pellet. Tubes were incubated on ice for 30 minutes to allow binding. Unbound antibody was removed by washing twice with flow cytometry buffer as previously described. Secondary antibody consisted of Alexa-Fluor 647 goat anti-human IgG (Invitrogen, UK) or Alexa-Fluor 488/647 goat anti-mouse IgG (Invitrogen, UK) for detection of the PGN401 and anti-EBOV antibodies, respectively. Secondary antibody was diluted at 1 : 400 with flow cytometry buffer containing 50% FCS, and 100 *μ*L of the appropriate antibody was added to each tube. Tubes were incubated on ice for 30 minutes to allow binding to occur. Unbound antibody was removed by washing twice with flow cytometry buffer as previously described. Cell pellets were fixed in flow cytometry fixation buffer that contained 4% paraformaldehyde (eBioscience, UK). Tubes were fumigated overnight with formalin vapor before removal from the CL4 laboratory. Samples were run on a Beckman Coulter FC500 flow cytometer and analyzed using Cytomics CXP software. Cells were gated using a forward scatter/side scatter density plot. Binding of Alexa Fluor 488 and 647 antibodies was determined by a histogram of fluorescence from the FL1 and FL4 channels, respectively. For dual color staining, a quadrant plot was created to identify single- and dual-labeled cells.

### 2.3. Immunofluorescence Assay

VeroE6 cells (European Collection of Cell Cultures, UK) were cultured on 8-well LabTek II chamber slides (Thermo Scientific Nunc, UK) and infected with EBOV for 5 days. Antibody preparations of PGN401 and a positive anti-EBOV monoclonal antibody control (clone FE25, Abcam, UK) were diluted with PBS to a concentration of 100 *μ*g/mL before 500 *μ*L was added to the relevant chambers of the slide. PBS alone and isotype antibodies (Erbitux and mouse IgG2a (Abcam, UK)) were used as negative controls. After 1 hour at 37°C the cells were washed 3 times by immersion in PBS and dried. For detection, Alexa Fluor 488-conjugated anti-mouse or anti-human antibody (Invitrogen, UK) was diluted at 1 : 400 and used at 500 *μ*L per well. After 1 hour at 37°C the cells were washed 3 times with PBS and dried. Slides were fixed with a 4% formaldehyde solution before removal from the Containment Level 4 laboratory. Cells were microscopically observed using an EVOS FL imaging system using a GFP imaging cube (Life Technologies, UK).

### 2.4. ELISA Assay

For coating with live EBOV virions, fluid from infected cultures were clarified by centrifugation at 400 ×g for 10 minutes. The supernatant fluid was transferred onto sucrose solution (20% sucrose in TH buffer) and particles were purified by ultracentrifugation at 25,500 rpm at 4°C in a SW28 rotor (Beckman Coulter, UK). The sucrose cushion and supernatant fluid was discarded and the pellet air-dried for 10 minutes before resuspending in 3 mL PBS. A 660 nm protein assay (Thermo Scientific Pierce, UK) was used to determine the protein concentration in the purified stocks. Stocks were diluted with PBS to a concentration of 10 *μ*g/mL and 100 *μ*L added to wells of a microplate (Immulon 1B, VWR, UK). For a virus negative control, PBS alone was added. Plates were left overnight at 4°C to allow binding to occur. For coating with PS, hexane solvent (Sigma, UK) was used to dilute PS to a concentration of 5 *μ*g/mL and 100 *μ*L added per well of the microplate. For an antigen negative control, hexane alone was added. Plates were incubated at room temperature in a fume cabinet until the hexane had evaporated and the plates were dry. To assess binding to EBOV glycoprotein, recombinant* Zaire ebolavirus* glycoprotein minus the transmembrane region (rZEBOV GPdTM, IBT Bioservices, US) was diluted to 1 *μ*g/mL with carbonate-bicarbonate buffer (Sigma, UK) and 100 *μ*L added per well of a microplate. To remove unbound antigen, plates were washed with five washes of 200 *μ*L PBS per well. To each well, 200 *μ*L of protein-free blocking buffer (PFBB, Pierce, UK) was added and incubated for 30 minutes at 37°C. Blocking buffer was removed by washing five times with PBS. Dilutions of PGN401 were made in low-binding microplates (Corning, UK) with binding buffer, consisting of 10% dialyzed FCS (Invitrogen, UK) in PBS, before 100 *μ*L was transferred across into the assay plate. Erbitux was used as a non-PS binding isotype control antibody [33]. Mouse anti-ZEBOV GP mAb (clone 4F3, IBT Bioservices, US) was used as a positive control for binding to the recombinant glycoprotein. Plates were incubated for 1-2 hours at 37°C to allow antibody binding to occur. Unbound antigen was removed by washing five times with PBS. Secondary antibody consisted of HRP-conjugated goat anti-human IgG (Jackson ImmunoResearch, USA) diluted at 1 : 2500 in binding buffer, with 100 *μ*L added per well. Plates were left for 1 hour at 37°C before unbound antibody was removed by washing five times with PBS. 3,3′,5,5′-Tetramethylbenzidine (TMB) substrate (Insight Biotechnology, UK) was added to each well at a volume of 100 *μ*L and left for 15 minutes at room temperature to allow the colorimetric reaction to occur. The reaction was stopped by addition of 100 *μ*L of 2 M H_2_SO_4_ (Fisher Scientific, UK). Absorbances were read by an automated plate spectrometer at a wavelength of 450 nm within 30 minutes of adding the stop solution and analyzed using SoftMax Pro software (Molecular Devices, UK). Data were plotted on a line graph as absorbance at 450 nm* versus* antibody concentration in a log ng/mL scale.

## 3. Results

### 3.1. PGN401 Specifically Binds to Cells Infected with EBOV

**Figure 1:**
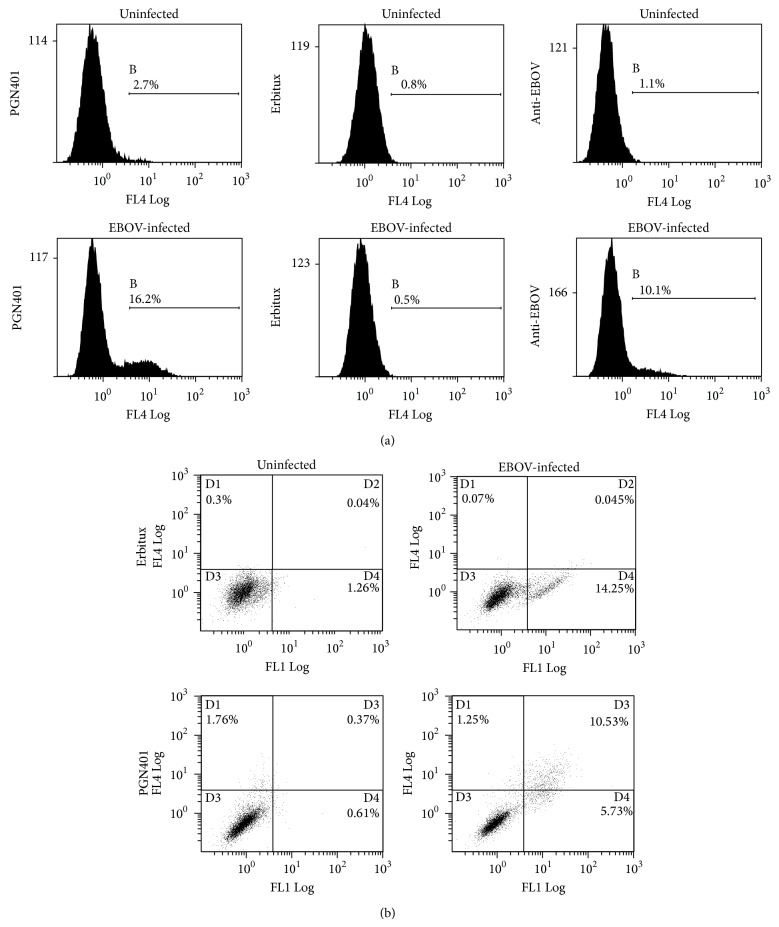
Flow cytometry staining of EBOV-infected cells. (a) Single color staining of cells with anti-EBOV, Erbitux, and PGN401 antibodies. Histograms show frequency of cells versus level of fluorescence intensity. The marker regions quantify the percentage of cells stained above background levels. (b) Dual color labeling with anti-EBOV and PGN401 antibodies. The *x*-axis relates to the detection of the FL1 channel that detects Alexa-Fluor 488 staining (anti-mouse detector for EBOV antibody) and the *y*-axis, the FL4 channel that detects Alexa Fluor 647 staining (anti-human detector for PGN401 and Erbitux). Quadplots identify the percentage of cells within each region.

**Figure 2:**
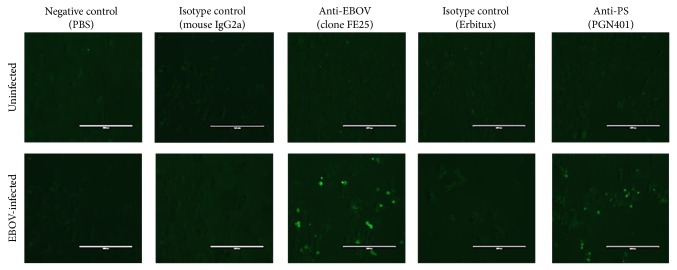
Immunofluorescence staining of uninfected and EBOV-infected cells after staining with antibodies against EBOV (clone FE25), PS (PGN401), and isotype antibodies. Scale bar indicates 200 nm.

VeroE6 cells that had been infected with Ebola Zaire virus at a multiplicity of infection (MOI) of approximately 0.5 for five days were used to determine recognition by PGN401 antibody. Staining with an anti-EBOV antibody (clone FE25) showed 9% of cells were specifically labeled (Figure 1(a)). With the PGN401 antibody, 13.5% of cells were specifically stained, compared with 0% for the Erbitux isotype control antibody. This result was repeatable, with a second experiment showing 21.4%, 21.1%, and 0% staining for anti-EBOV, PGN401, and Erbitux, respectively. Dual-colour labeling was used to determine whether the same cells that were stained with the anti-EBOV antibody were also those that PGN401 bound. Results demonstrated that the PGN401 bound to cells to which anti-EBOV antibody was also binding (Figure 1(b)). To support this observation, immunofluorescence testing was conducted using cells infected with EBOV. Results showed specific binding to EBOV-infected cells by the PGN401 and positive control anti-EBOV antibodies with no staining observed with the negative control and isotype antibodies (Figure 2). The immunofluorescence assay was run on two separate occasions and successfully demonstrated that the results were repeatable.

### 3.2. PGN401 Binds to Purified Ebola Zaire Virions

**Figure 3:**
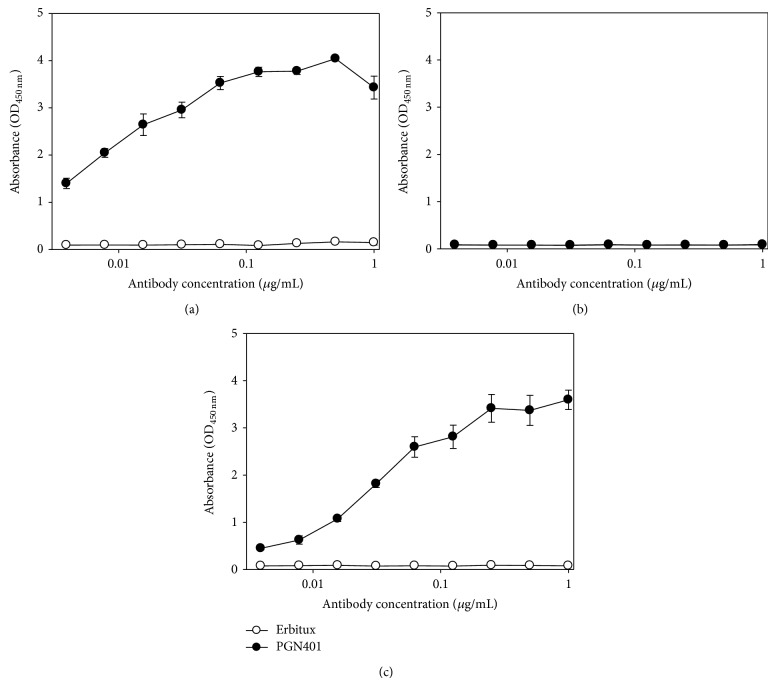
Binding of Erbitux and PGN401 antibodies to plates coated with (a) EBOV, (b) PBS, or (c) PS antigen using a twofold dilution series starting at 1 *μ*g/mL. Binding levels were assessed by measurement of absorbance at a wavelength of 450 nm. Data points show mean values from three replicate wells with error bars denoting standard error.

**Figure 4:**
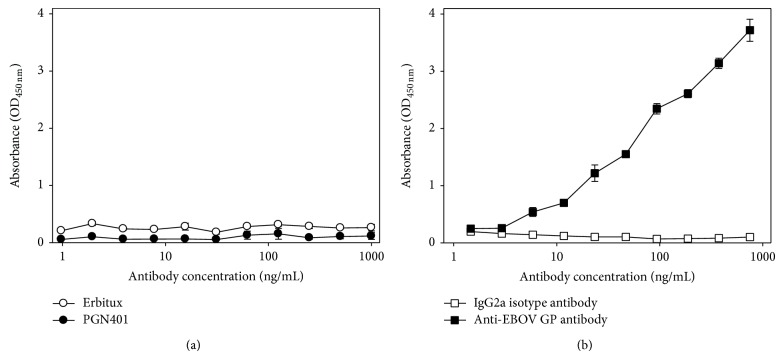
Binding of antibodies to recombinant EBOV glycoprotein. (a) Erbitux and PGN401 antibodies. (b) Polyclonal anti-EBOV glycoprotein antibody and IgG2a isotype control. Data points show mean values from three replicate wells with error bars denoting standard error.

Concentrated EBOV was used as an antigen to evaluate binding of PGN401 directly to the virus. A quantitative assay on the ultracentrifuged stocks demonstrated an increase in viral titre of approximately 4 logs, from 10^7.73^ in the unconcentrated stock to 10^11.6^ TCID_50_/mL. A protein quantification assay demonstrated that the concentrated stock contained 620 *μ*g/mL which was then diluted for use in the ELISA studies. Results showed that PGN401 bound specifically to EBOV virus, with no background binding to PBS buffer alone (Figures 3(a) and 3(b)). Under conditions of antigen excess, PGN401 recognition of EBOV in ELISA was equivalent to monoclonal antibody recognition of PS (Figure 3(c)). A further ELISA experiment was conducted using recombinant EBOV glycoprotein to ascertain whether the PGN401 recognized this viral protein. Results showed that PGN401 did not bind to the glycoprotein (Figure 4(a)) whereas the positive control antibody did (Figure 4(b)). All ELISA experiments were repeated at least twice in order to confirm the results achieved.

## 4. Discussion

The data presented in this study clearly demonstrate that PGN401 has strong binding properties for EBOV virions and EBOV-infected cells. The attachment of the antibody to EBOV virions is indicative of PS being present on the EBOV membrane. Previous studies have shown that PS enhances receptor independent virus entry mechanisms [34]. Additionally, PS found on the surface of the vaccinia virus membrane has been shown to trigger the signaling, blebbing, and macropinocytic event, suggesting that the virus uses an entry mechanism based on mimicry of apoptotic bodies [35]. Apoptotic mimicry provides a route for virus uptake and may also help in the evasion of immune recognition; it has also been observed for hepatitis B virus [36], Lassa fever virus [23], and HIV-1 [37]. Entry via macropinocytosis offers the advantage to the virus of not being exposed to the full range of the immune system, thus delaying immune recognition of the infected cells [35]. In addition, macropinocytic entry gives viruses the mechanisms to broaden their host range and tissue specificity [35]. Many enveloped viruses that bud out from the plasma membrane are assembled in, and bud out of, lipid rafts [38]. This includes EBOV [39]. The lipid rafts are enriched with PS [40] and are structurally modified during apoptosis and are involved with the externalization of PS [41]. Other viruses that are believed to egress from rafts include HIV-1 [42], influenza A virus [43], vesicular stomatitis virus [44], Marburg virus [39], and respiratory syncytial virus [45]. Previous studies have also documented that macropinocytosis is used by EBOV for cell entry [46–48]. Therefore, our results further confirm these findings.

The current studies utilized the VeroE6 cell line as a simple, straightforward, and permissive host* in vitro*. To test for PGN401 binding, we chose the furthest time point from infection before cytopathic effect was observed in order to allow reasonable time for PS to become exposed on the cell surface. Previous studies have shown induction of PS translocation occurs relatively soon after apoptosis [49]. In follow-on studies we aim to detail these findings with the use of primary cells such as macrophages and dendritic cells which are natural targets for EBOV infection [50, 51]; these will also provide the opportunity to specify the postinfection time points when PS becomes exposed on these natural cellular targets. We have assessed the binding of PGN401 independently using two different techniques, flow cytometry, and immunofluorescence. However, the binding of PGN401 to purified virions has only been conducted by ELISA and we are not able to rule out nonspecific binding to impurities in the virus preparation. Nevertheless, this material was purified through a 20% sucrose cushion and there was no evidence of sample degradation which often accompanies the coconcentration of other proteins. Thus we are confident that PGN401 binds PS of EBOV virions.

The mechanism of action of PGN401 is not fully understood. In the elimination of Pichindé virus infection and viremia in the guinea pig model, PGN401 is thought to function via at least two different mechanisms. Firstly, it caused opsonization and clearance of infectious virus from the bloodstream, leaving less virus to infect other tissues and secondly, it induces ADCC of virus infected cells [23]. Other mechanisms by which an antibody may neutralize pathogenic material can include antibody-dependent cellular phagocytosis, complement-dependent cytotoxicity, opsonization, and steric hindrance of ligand activity, almost all of which require the antibody Fc region to interact with cellular receptors [52, 53]. Triggering via the Fc receptor of IgG has also been reported to stimulate chemokine release from natural killer cells, monocytes, and dendritic cells [54–56]. The efficiency of PGN401 may be enhanced by adding extra target molecules to the antibody. For example, adding interleukin (IL)-2 has been shown to enhance immunogenicity of a PS-targeting antibody for use as a breast cancer vaccine [57]. Another option may be to use the antibodies to deliver radionuclides that emanate lethal doses of cytotoxic radiation to target cells [58].

This study has shown that the PS-targeting antibody, PGN401, binds to EBOV-infected cells and to purified EBOV virions. Due to PGN401 having been used for several human clinical trials in cancer, from Phase I to III [13, 20, 21], its repurposing of use for filovirus therapy through licensure or emergency use may present an attractive option. Due to the anticipated small market size of any antifiloviral treatment, the use of therapies primarily designed for other conditions confers several advantages in reducing the costs of bringing an effective treatment to clinical use due to having already negotiated some of the steps required for regulatory approval. Future work to determine whether this approach elucidates any protective effect using* in vivo *models of EBOV infection is planned.
